# Transcriptome landscape comparison of periodontium in developmental and renewal stages

**DOI:** 10.3389/fendo.2023.1154931

**Published:** 2023-03-15

**Authors:** Yuwei Deng, Nan Luo, Ming Xie, Ling He, Ruixue Jiang, Nan Hu, Jin Wen, Xinquan Jiang

**Affiliations:** ^1^ Shanghai Engineering Research Center of Advanced Dental Technology and Materials, Shanghai Key Laboratory of Stomatology & Shanghai Research Institute of Stomatology, National Clinical Research Center for Oral Diseases, Shanghai Ninth People’s Hospital, College of Stomatology, Shanghai Jiao Tong University School of Medicine, Shanghai, China; ^2^ Department of Prosthodontics, Ninth People’s Hospital Affiliated to Shanghai Jiao Tong University School of Medicine, Shanghai, China; ^3^ Department of Preventive Dentistry, Ninth People’s Hospital, School of Medicine, Shanghai Jiao Tong University, Shanghai, China; ^4^ Department of Radiation Oncology, David Geffen School of Medicine at University of California, Los Angeles (UCLA), Los Angeles, CA, United States; ^5^ Department of Endodontics, Ninth People’ Hospital, School of Medicine, Shanghai Jiao Tong University, Shanghai, China

**Keywords:** periodontium, bioinformatics, regeneration, signal, tissue microenvironment

## Abstract

**Objectives:**

Periodontium regeneration remains a significant challenge in clinics and research, and it is essential to understand the stage-specific biological process in situ. However, differing findings have been reported, and the mechanism has yet to be elucidated. The periodontium of adult mice molars is considered to be stable remodeling tissue. At the same time, the continuously growing incisors and the developing dental follicle (DF) of postnatal mice highly represent fast remodeling tissue. In this study, we attempted to explore different clues of temporal and spatial comparisons to provide improved references for periodontal regeneration.

**Methods:**

Periodontal tissues from the developing periodontium (DeP) of postnatal mice, and continuously growing periodontium (CgP) and stable remodeling periodontium (ReP) of adult mice were isolated and compared using RNA sequencing. Based on the Dep and CgP separately compared with the ReP, differentially expressed genes and signaling pathways were analyzed using GO, KEGG databases, and Ingenuity Pathway Analysis (IPA). The results and validation were obtained by immunofluorescence staining and RT-PCR assays. Data were expressed as means ± standard deviation (SD) and analyzed by GraphPad Prism 8 software package, and one-way ANOVA was used to test multiple groups.

**Results:**

Principal component analysis showed that the three groups of periodontal tissue were successfully isolated and had distinct expression profiles. A total of 792 and 612 DEGs were identified in the DeP and CgP groups compared with the ReP. Upregulated DEGs in the DeP were closely related to developmental processes, while the CgP showed significantly enhanced cellular energy metabolism. The DeP and CgP showed a common downregulation of the immune response, with activation, migration, and recruitment of immune cells. IPA and further validation jointly suggested that the MyD88/p38 MAPK pathway played an essential regulatory role in periodontium remodeling.

**Conclusion:**

Tissue development, energy metabolism, and immune response were critical regulatory processes during periodontal remodeling. Developmental and adult stages of periodontal remodeling showed different expression patterns. These results contribute to a deeper understanding of periodontal development and remodeling and may provide references for periodontal regeneration.

## 1 Introduction

The periodontium, composed of the gingiva, cementum, periodontal ligament, and alveolar bone, plays an essential role in supporting and maintaining tooth function. Although studies have shown that the periodontium contains a population of stem cells that can further differentiate into fibroblasts, cementoblasts, osteoblasts, and osteoclasts, with the ability to self-renew and remodel (1), it is still highly challenging to repair periodontal defects caused by trauma and congenital malformation. A unique characteristic of the periodontal complex (cement–periodontal ligament–bone) is the ordered hierarchical structure within its tiny space, posing spatiotemporal compartmentalization as a critical requirement for micron-scale regeneration and functional restoration of multiple tissues (2). However, current clinical methods, such as guided tissue regeneration, still cannot effectively prevent disorganized or spontaneous tissue formation that can lead to anomalies, such as ankyloses (3). It has gradually become a consensus that periodontal regeneration based on the biological rationales of *in situ* remodeling to promote orderly tissue patterns and morphogenesis could be more effective.

Recently, researchers have employed new techniques and methods to help obtain more information on tissues and cells. Biomedical “omics” approaches rely on collecting large amounts of omics data, combined with bioinformatics and biostatistics, to drive new and deeper insights into complex biology (4). In previous studies, single-cell RNA-seq analysis was successfully applied to analyze the cellular heterogeneity of cells in the periodontium and their developmental relationship with early precursor cells, such as identifying PTHrP+ cells in the dental follicle as important precursor cells that may give birth to osteoblasts, periodontal ligament cells, and cementoblasts to provide a cell source for later periodontal tissue formation (5). Some transcriptome studies have investigated the gene expression patterns of periodontal ligament cells under specific culture conditions, such as mechanical loading and osteogenic induction, to clarify the mechanism of different environmental factors stimulating the differentiation process (6, 7). Furthermore, deepening the understanding of oral proteomics and the microbiome provides meaningful clues for detecting the onset of periodontal diseases, improving treatment effects, and promoting personalized treatment (8, 9). However, the remodeling and repair of periodontal tissues result from complex cell–environment and cell-cell interactions. Detecting *in vitro* periodontal cells or specific cell populations is insufficient for systematically understanding periodontal remodeling. The process of how periodontal tissue characteristics regulate periodontal remodeling *in situ* in a healthy state has not been elucidated. Therefore, sample sources for omics exploration should be further considered to fully understand the biological process of dynamic periodontal tissue remodeling and the cellular and molecular mechanisms controlling tissue patterns and morphogenesis that contribute to the development of periodontal regeneration and self-repair methods.

Regeneration shares many mechanisms with embryonic development. However, renewal to restore original tissue structure and biological function does not appear equivalent to developmental events. Adult tissues and organs do not necessarily show the same gene expression profiles as their embryonic, fetal, or juvenile counterparts (10). For example, Driskell’s team compared genes and cells in developing and adult skin and found that a transcription factor, *Lef1*, which controls hair follicle formation during development, acts as a molecular switch in the skin of young mice. It is essentially turned off after skin formation, but when specific cells in adult mice were activated, their skin healed a wound without leaving a scar (11). From another aspect, although the regenerative capacity of most tissues and organs declines with age, some organs continue to grow and remodel in adulthood, providing promising clues for treating adult patients. For example, studies have shown that the continuous activation of Wnt signaling may play a role in the growth and self-renewal of the kidney, providing a therapeutic target for promoting or restoring the regenerative capacity of the kidney (12). Thus, research on tissue regeneration should be conducted throughout the life cycle. Since the developmental stages in youth and adult regeneration processes are not identical, exploring periodontal remodeling in youths and adults is equally essential for periodontium regeneration. Thus, studying the different stages of active remodeling of the periodontium is necessary.

Periodontium develops from the dental follicle. Accompanied by root development and tooth eruption, dental follicles differentiate to form periodontal tissues (13). At this stage, the follicle coordinates the extensive formation and remodeling of the extracellular matrix. It is worth noting that rodent incisors continue to grow throughout life. Compared with molars, the incisors have sites of more active remodeling of the extracellular matrix (14), which provides us with an ideal model for studying periodontal renewal and remodeling in adult individuals.

In this study, mouse dental follicles and incisor periodontium were used as representative tissues representing actively remodeling periodontal tissue in the developmental and adult stages. They were compared with the stable remodeling periodontium of molar teeth. Genome-wide mRNA expression profiling and in-depth bioinformatics analysis were performed to characterize the regulation of gene expression in the actively remodeling periodontal tissues. Attention was paid to the shared or unique signaling regulatory mechanisms during periodontal remodeling in the developmental and maturation stages. More importantly, multi-directional regulatory targets in various processes of periodontal tissue remodeling were proposed to provide a basis for a more comprehensive understanding of the underlying mechanism and the promotion of periodontal defect repair.

## 2 Methods

### 2.1 Tissue acquisition and preparation

**Figure 1:**
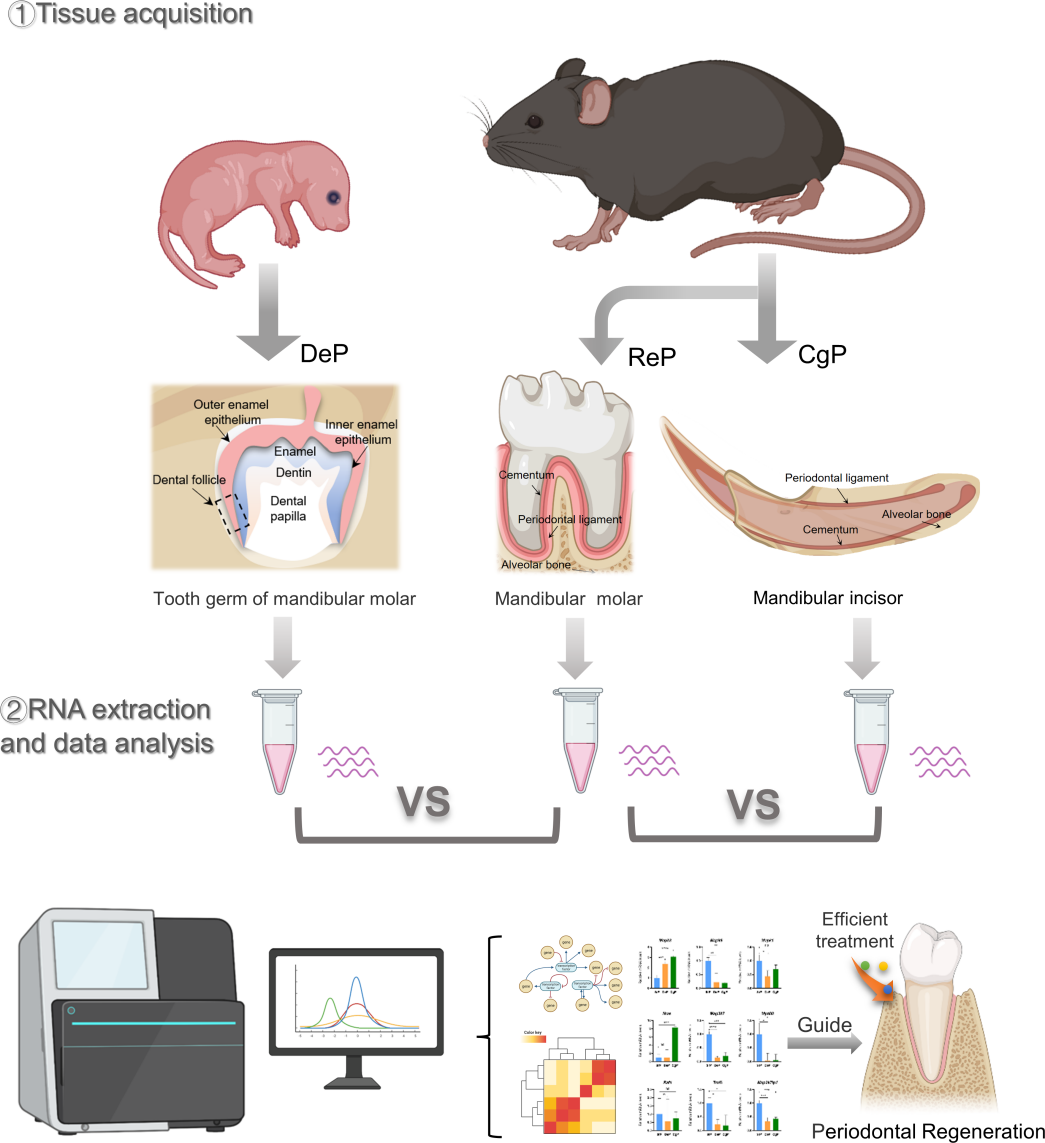
Schematic representation of the research.

All of the experimental animal procedures were approved by the Laboratory Animal Ethics Committee at the Ninth People’s Hospital affiliated to Shanghai Jiao Tong University School of Medicine. C57 mice were used in this study. The molar tooth germs were excised from postnatal day 3 mice under a zoom stereo microscope (Olympus SZ51, Tokyo, Japan) by cutting the tooth capsule open and carefully removing the tooth papilla. Then, 1/3 of the dental follicles adjacent to the root were peeled off. The periodontal ligament of the mandibular molar and the lingual periodontal ligament of the mandibular incisor were dissected from eight-week-old adult mice. Each dental follicle sample contained tissue from eight to ten mice. Each molar and incisor periodontium sample contained tissue from three mice. There were three samples of each group, and the tissues were stored in a tissue storage solution for RNA extraction. In addition, the tissue of three mice in each group was obtained for histological experiments. **Figure 1** illustrates the flow diagram of this study.

### 2.2 Histological analysis

The mandibles of postnatal and adult mice (n = 3) were dissected and fixed in 4% paraformaldehyde. Mandibular bones were subsequently demineralized in 0.5 M ethylenediaminetetraacetic acid. The tissue was embedded in paraffin and cut into 5-μm tissue sections.

For hematoxylin and eosin (H&E) staining, nuclei were stained with Gill’s hematoxylin, then stained blue with diluted ammonium hydroxide, and the cytoplasm was counterstained with acidified eosin. Immunofluorescent staining was performed according to a standard procedure, and 4,6-diamidino-2-phenylindole (DAPI) was used to visualize the nuclei. Mean fluorescence intensity was calculated for three fields of tissue for each group to indicate cell density using ImageJ software.

### 2.3 RNA preparation and sequencing

Total RNA was isolated from each sample using an RNA mini kit (Qiagen, Germany). RNA quality was examined using gel electrophoresis and a Qubit fluorometer (Thermo, Waltham, MA, USA). For RNA sequencing, strand-specific libraries were constructed using a TruSeq RNA sample preparation kit (Illumina, San Diego, CA, USA), and sequencing was carried out using an Illumina Novaseq 6000 instrument.

### 2.4 Real-time PCR analysis of gene expression

Total cellular RNA of periodontal tissues was extracted with TRIzol reagent (Invitrogen; Thermo Fisher Scientific, Inc.), and reverse transcription was performed using a PrimeScript RT reagent kit (Takara Bio, Inc., Otsu, Japan). Gene-specific primers were synthesized commercially (Shenggong Co., Ltd., Shanghai, China), and their sequences are listed in **Table 1**. In one reaction, a 10 μL SYBR Premix Ex Taq kit (Takara Bio, Inc.) was used to amplify 1 μL of cDNA (mixed with 8 μL of distilled water and 0.5 μL of each primer). A Bio-Rad iQ5 real-time PCR system (Bio-Rad Laboratories, Inc., Hercules, CA, USA) was then used to detect gene expression. All of the relative gene expression values were normalized to *Actb* (encoding *β-actin*) based on the 2^ΔΔ^Cq method.

**Table 1:** Sequences of the primers used for a reverse transcription-quantitative polymerase chain reaction.

Genes	Forward primer	Reverse primer
*Acta1*	TGAAGTGTGACGTGGACATC	GGAGGAGCAATGATCTTGAT
*Mapk3*	CAAGGAGCGGCTGAAGGAGTTG	GAAGGAGCAGGTAGGAGCAGGAC
*Raf1*	GCTTCCCTACGCCCACATCAAC	CACAGTCAGCCACCAACCTCTTC
*Mapk1*	GCCTTCCAACCTCCTGCTGAAC	CGTACTCTGTCAAGAACCCTGTGTG
*Nras*	GTGGTTGGAGCAGGTGGTGTTG	AGTATGTCCAGCAGGCAGGTCTC
*Mapk8*	CACAGTGAGCAGAGCAGGCATAG	TTGTCAGGAGCAGCACCATTCTTAC
*Map3k7*	CTCGTCCTCCTCCTCGTCTTCTG	TCTTCCGACAACCTCTTCCACCTC
*Traf6*	ACAGCAACTCTTACAGCCAGGAAAC	AACCACTGAGCCAATTCTCCAACC
*Myd88*	AGCAGAACCAGGAGTCCGAGAAG	GGGCAGTAGCAGATAAAGGCATCG
*Map3k7ip1*	GTCGTGGCAGTCCTTCTCAACAG	TCGTCCTCGTTCTCGGTGGTG

### 2.5 RNA-seq and identification of differentially expressed genes

The sequencing data have been deposited in the NCBI Sequence Read Archive (SRA) database under the accession code PRJNA928506. The raw data were processed by Skewer, and data quality was checked by FastQC v0.11.2 (http://www.bioinformatics.babraham.ac.uk/projects/fastqc/). The expression of the genes was calculated by fragments per kilobase of exon model per million mapped reads (FPKM) using Perl. Differentially expressed genes (DEGs) between different tissues were determined using the MA-plot-based method with random sampling (MARS) model in the DEGseq package. The thresholds for determining DEGs were q value (adjusted p-value) < 0.05 and absolute fold change ≥ 1.5.

### 2.6 Bioinformatics analysis

Venn diagram and principal component analyses were performed using R software. DEGs were chosen for function and signaling pathway enrichment analysis using GO and KEGG databases to discover potential regulatory networks. The significantly enriched pathways were determined when p < 0.05.

The Ingenuity Pathway Analysis system (IPA system; Qiagen China Co., Ltd.), which includes canonical pathway analysis, disease and function, regulator effects, upstream regulators, and molecular networks, was also used for subsequent bioinformatics analysis. For each analysis, a p-value < 0.05 was set as the threshold.

### 2.7 Statistical analysis

GraphPad Prism 8 software package was used for statistical analysis. Data were expressed as means ± standard deviation (SD), and one-way ANOVA was used to test multiple groups. A statistically significant difference was considered at *p < 0.05, **p < 0.01, ***p < 0.001, and ****p < 0.0001.

## 3 Results

### 3.1 Acquisition of mice periodontal tissue

**Figure 2:**
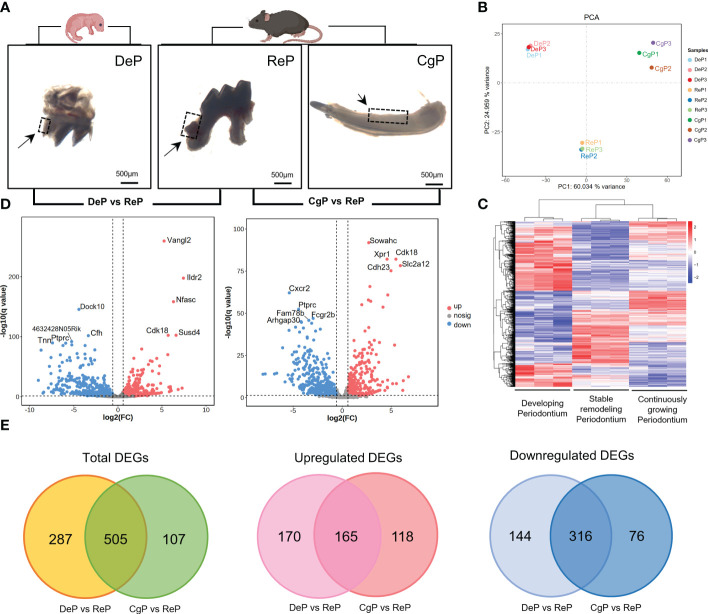
Comprehensive analysis of DEGs. **(A)** Postnatal mice dental follicles, adult mice incisors, and molars periodontal tissues were obtained. Tissues were collected around the tooth germ/tooth(inside the black dotted line). **(B)** Principal component analysis of the three groups. The higher the variation in gene expression patterns across samples was, the greater the distance among samples. **(C)** Heat map of differential gene expression levels in different periodontal tissues. The color saturation in the heat map indicates the expression of genes. Blue shows the lowest expression of genes, and red indicates the highest expression of genes. **(D)** Comparison of DEGs in DeP/CgP versus ReP using the Volcano plot. Red dots indicate significantly upregulated genes, blue dots indicate downregulated genes considerably, and gray dots represent nonsignificantly differentially expressed genes. **(E)** Venn diagram. DEGs were identified as having a q value (adjusted p-value) of < 0.05 and |fold change| of ≥ 1.5.

Three days after birth, the mouse molars developed to the late bell stage and had not yet erupted. The periodontal part of the dental follicles would develop into the periodontium. In 8-week-old mice, the teeth had erupted completely and participated in chewing and other maxillofacial activities. The molar dental follicle (DeP), the molar periodontium (ReP), and the lingual periodontium of the mandibular incisors (CgP) were collected, as shown in **Figure 2A**, and were subjected to high-throughput sequencing using the Illumina Hiseq platform.

The transcript abundance of each gene was estimated by FPKM. Principal component analysis was performed to determine whether the expression profiles obtained for each group represented unique molecular features suggestive of developing or actively remodeling periodontal tissues. The results showed that principal component 1 (PC1) accounted for 60.034% of the variance. Principal component 2 (PC2), accounting for 24.959% of the variance, clearly separated the three groups in this study (**Figure 2B**). **Figure 2C** shows the heat map after the bidirectional hierarchical clustering of the genes and samples, which sufficiently demonstrated the intra-group consistency of the groups, and the inter-group difference was apparent.

### 3.2 Significant changes in gene expression were observed in DeP and CgP compared with the ReP

Significant DEGs were identified with a significance threshold of |fold change| ≥ 1.5 and q value (adjusted p-value) < 0.05. Identification of DEGs revealed different gene expression regulations of the remodeling process in DePs and CgPs. When the DeP was compared with the ReP (DeP versus ReP comparison), 335 genes were found to be upregulated, and 460 were downregulated. The top five upregulated genes were *Vangl2*, *Ildr2*, *Nfasc*, *Susd4*, and *Cdk18*, while the top five downregulated genes were *Dock10*, *Cfh*, *4632428N05Rik*, *Tnn*, and *Ptprc*, as shown in **Figure 2D** (left). A comparison of the CgP and ReP (CgP versus ReP comparison) revealed 283 upregulated genes and 392 downregulated genes. The top five upregulated genes were *Sowahc*, *Cdk18*, *Xpr1*, *Slc2a12*, and *Cdh23*, while the top five downregulated genes were *Cxcr2*, *Ptprc*, *Fcgr2b*, *Fam78b*, and *Arhgap30*. A volcano plot based on DEGs is shown in **Figure 2D** (right). Detailed information on the DEGs is provided in **Supplementary Tables 1**, **2**.

To further compare the guiding significance of the DeP and CgP samples for the regeneration and repair of the periodontium, we evaluated the DEGs of these two comparisons. A Venn diagram (**Figure 2E**) revealed the two overlapping and unique DEGs compared with the ReP. The DeP and CgP shared 165 upregulated DEGs and 316 downregulated DEGs. At the same time, 170 and 118 upregulated DEGs and 144 and 76 downregulated DEGs were unique for the DeP and CgP, respectively. The results suggested that the gene regulation of periodontal tissue remodeling processes in developing and adult individuals was generally similar but still had different properties.

### 3.3 DeP and CgP were characterized by enhanced tissue development and cellular energy metabolic activity, respectively

**Figure 3:**
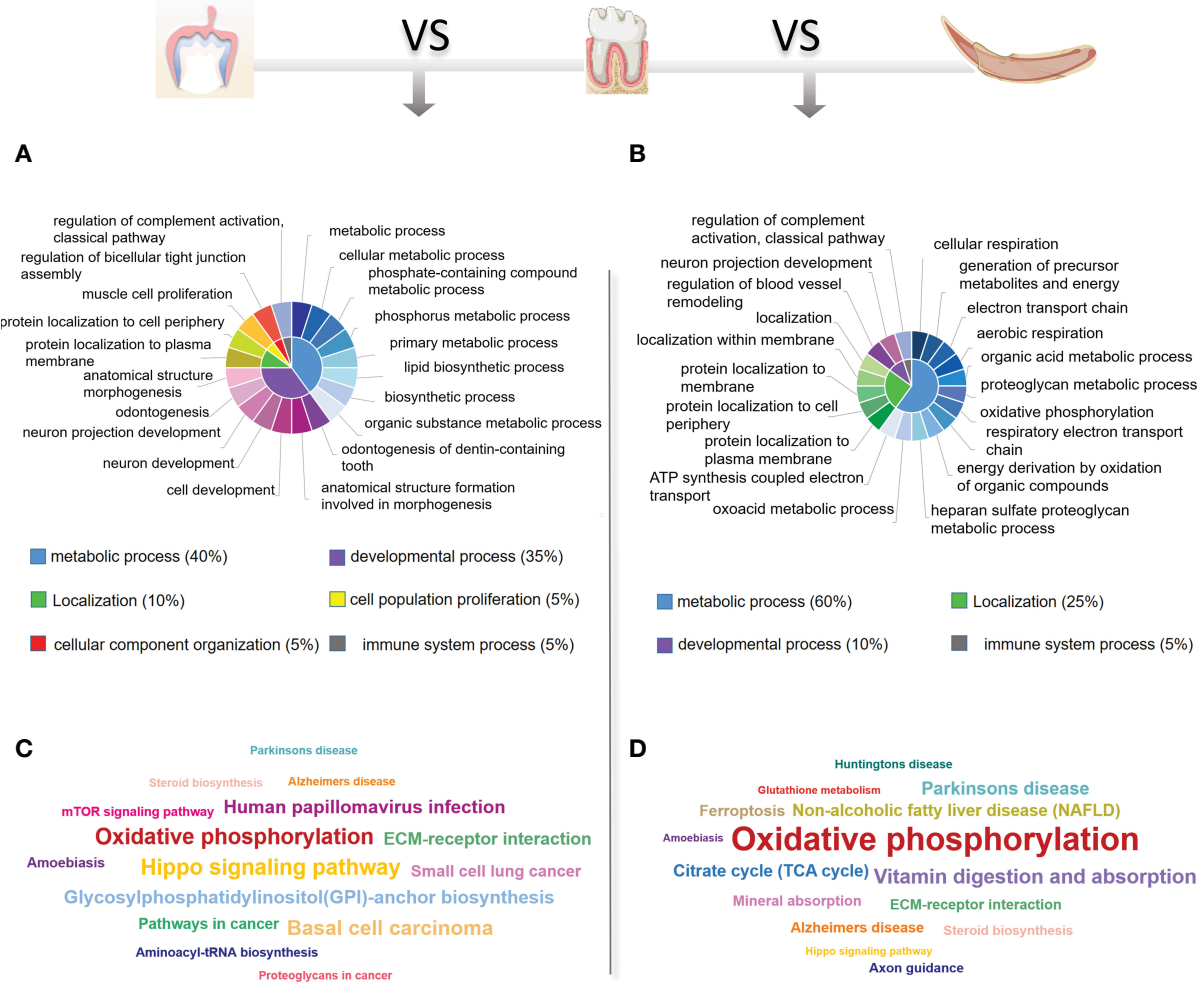
GO and KEGG database enrichment analyses were used to determine the critical pathways of the upregulated DEGs in DeP and CgP. **(A, B)** The top 20 biological processes in the GO enrichment analyses of the upregulated DEGs in DeP **(A)** and CgP **(B)**. The same color in the inner circle represents the same category of pathways in the outer ring, and the area size of the inner circle represents the proportion of the pathway category. **(C, D)** The top 15 pathways in the KEGG enrichment analyses of the upregulated DEGs in DeP **(C)** and CgP **(D)**. The size of the word in the word cloud is proportional to the -log10 (p-value).

To identify the critical physiological mechanisms involved in periodontium remodeling, enrichment analyses of DEGs in the DeP and CgP versus the ReP were performed. In GO analysis, upregulated genes in the DeP versus ReP comparison were mainly enriched in pathways involved in cell development (odontogenesis of dentin-containing tooth, odontogenesis, neuron development, and neuron projection development). Among the processes related to cell metabolism, it was noticed that the metabolism of phosphate-containing compounds metabolic process and phosphorus metabolic process were particularly prominent. **Figure 3A** illustrates the top 20 biological processes enriched by upregulated DEGs of DeP versus ReP. The upregulated DEGs in the CgP versus ReP comparison showed significant enrichment in the cellular energy metabolism process. In particular, aerobic metabolism accounted for a high proportion of energy metabolism (aerobic respiration, oxidative phosphorylation, and energy derivation by oxidation of organic compounds).

Moreover, the significant enrichment of extracellular matrix metabolic processes suggested that they may be involved in active tissue remodeling processes, such as the proteoglycan metabolic process and the heparan sulfate proteoglycan metabolic process (**Figure 3B**). As shown by the KEGG analysis (**Figures 3C, D**), although the upregulated DEGs in DeP and CgP tissues were both enriched in the oxidative phosphorylation pathway, the Hippo signaling pathway, which is closely related to dental development, was more significantly enriched in the DeP. Correspondingly, the citrate cycle (TCA cycle) and other energy metabolism pathways in the CgP were enhanced.

The IPA system was used to analyze the critical molecular networks involved in periodontium remodeling. The interaction network analysis was used to identify the interactions between molecules in the dataset and their related diseases and functions. Using the score value to rank all of the networks, it was found that the highest-ranked network in the DeP (score = 51) was associated with “connective tissue disorders” and involved in the disease regulation of “developmental disorders”. The results suggest that the gene expression changes in the DeP were closely related to the developmental process of the tissue. This network identified 32 molecules in the DEGs, which, together with the other three predicted molecules, constituted the corresponding molecular network, as shown in **Table 2**. The DEGs network affecting “connective tissue development and function” in the CgP also affected “carbohydrate metabolism”. These results suggested that the energy supply of cells plays a vital role in regulating the state of adult periodontal tissue. This network ranked 14th (score=24) and involved 19 molecules in the DEGs, which, together with 16 other predicted molecules, constituted the corresponding molecular network, as shown in **Table 3**. These results were consistent with GO and KEGG analyses, suggesting that the enhanced biological functions related to tissue development and energy metabolism were closely related to the formation and remodeling of periodontal tissues and may represent the characteristic cellular behavior of developing and mature periodontal tissues.

**Table 2:** A list of molecular networks of DEGs in Dep versus ReP.

ID	Molecules in Network	Score	Focus Molecules	Top Diseases and Functions
1	AHI1, ASPM, ATPase, C1orf115, C1orf226, CISD1, DTL, ECHDC1, EDEM3, ENAH, Erm, FRK, FYN, GMPPA, HERC4, KIF14, LTV1, MTHFD1L, NCKAP5, NSL1, NUF2, Pglycoprotein, PCBP3, PI15, PTBP1, QRSL1, RABGAP1L, RNPEP, SLC40A1, SMYD2, SPECC1L, SPTA1, TNNT2, TOR3A, ZBTB2	51	32	Connective Tissue Disorders, Developmental Disorder, Hereditary Disorder
2	ATP1B1, BAG2, BARD1, CDC73, CENPF, DHX9, DNA damage response, HDAC2, HDLBP, Histone h2a, HJURP, IGSF8, LGR6, LSS, MAP7, PTEFb, PARP1, PERP, PHLPP1, PRRC2C, R3HDM1, RB1CC1, SERPINB11, SLC39A10, SMG7, SP110, SP140, SPEG, TERF1, TMEM259, TRIP12, TUBA4A, UBE2T, XRCC5, ZDBF2	51	32	Cancer, Hereditary Disorder, Organismal Injury and Abnormalities
3	ACTR1B, Alpha tubulin, ALPI, CK1, CNST, COPA, CTDSP1, E130307A14Rik, EPRS1, FBXO30, G0S2, GJA1, GTF3C3, GTF3C6, HK1, IARS2, ILDR2, ILVBL, Insulin, KIF1A, MPC2, MYO1B, NCSTN, NGEF, PER2, PEX19, PEX3, PLEKHM3, RNF126, SLC30A10, STAU2, Synaptotagmin, SYT14, UBXN4, UGGT1	48	31	Cellular Assembly and Organization, Cellular Function and Maintenance, Hepatocellular Peroxisome Proliferation
4	ARPC5, ASCC3, BCR (complex), CHPF, COL6A3, CYB5R1, DNAJB2, FAM135A, Fcgr2, IFI16, IFNGR1, Interferon alpha, KLHDC8A, LPGAT1, MHC Class II (complex), NAB1, NEK7, PIGN, RGPD4 (includes others), RNASEL, Rnr, SEC63, SLAMF7, SLAMF9, SLC11A1, SLC19A1, STX7, SYNE1, TMEM200A, TMEM9, TOR1AIP1, TRAF3IP3, TSPAN15, VANGL2, WDR12	46	30	Cancer, Endocrine System Disorders, Organismal Injury and Abnormalities
5	ANK3, Cd24a, CD84, Ciap, DIS3L2, EDAR, elastase, HIVEP2, HLX, IGF receptor, Il12 receptor, LAMA2, Laminin2, LIMS1, LY9, LY96, Madcam1, MPZ, MYOC, NFASC, Nfatc, NFkB (complex), NUAK2, PBLD, RAPH1, RGS17, RNF152, SERPINB10, SH2D1B, ST18, STRADB, TTLL4, UNC5B, voltage-gated sodium channel, WNT10A	39	27	Cellular Assembly and Organization, Cellular Function and Maintenance, Nervous System Development and Function
6	AGFG1, Akt, ANGPTL1, CHIT1, CNNM4, Collagen type VI, CREG1, DIP2A, DNA-PK, FCGR1A/2A/3A, Fgf, FGFR, FMN2, GGT5, INPP4A, JPH1, MIER2, mir-205, N-Cadherin, NUS1, NYAP2, PID1, Pki, PREX2, Rab11, RUFY4, SCYL3, SEC16B, SLC9A2, SULF1, TBC1D8, TGFBRAP1, TMCC2, TRAPPC10, TRDN	37	26	Cell Morphology, Organ Morphology, Organismal Injury and Abnormalities
7	ADAMTS4, AGPAT3, Alpha 1 antitrypsin, ATIC, BMPR2, CEP85L, DES, ECEL1, ELF3, FN1, GADD45, KCNQ5, KIF21B, LATS1, LDL-cholesterol, LMAN2L, MIA3, MPZL1, MYL1, NEU2, Osteocalcin, Pak, Pias, PYCR2, ROCK, SERINC1, SUSD2, TAGLN2, TNS1, Top2, TWIST2, UTRN, VLDL-cholesterol, VSIG8, XPR1	37	26	Cardiac Arrythmia, Cardiovascular Disease, Hereditary Disorder
8	ABL2, ARHGAP18, BPNT1, Ecm, EFHC1, EGLN, ELANE, FAM117B, Fibrin, GREM2, growth factor, IGFBP5, ING5, L3MBTL3, MSC, NADH dehydrogenase, NADPH oxidase, NEURL3, NHLH1, NHSL1, NPL, NRP2, Par, PRSS57, PRTN3, SCML4, Serine Protease, SERPINE2, SLC30A1, Srgn, ST8SIA4, TMEM169, VASH2, Vegf, WASF1	37	26	Cell-To-Cell Signaling and Interaction, Dental Disease, Dermatological Diseases and Conditions
9	C1orf21, Cbp/p300, CENPL, Cox5b/Gm34962, EFHD1, ESR1, FAM184A, FARP2, FBXO28, GPC1, GULP1, INO80D, Integrinα, LAMB3, LAMC2, Laminin5, MICAL1, N-cor, NENF, PLEKHG1, Plexin A, PLXNA2, PNKD, Rar, RGL1, Smad, Sox, SOX13, SOX17, SUSD4, TCF/LEF, thymidine kinase, TPD52L1, UCK2, ZBTB18	35	25	Connective Tissue Disorders, Dermatological Diseases and Conditions, Developmental Disorder
10	Ap2, ARL4C, atypical protein kinase C, BVES, C2CD4C, C8orf44-SGK3/SGK3, COL4A4, COP I, DDR2, DNER, DOCK10, F11R, GTPase, INHA, KCTD3, LATS, Na, K -ATPase, NAV1, NPPC, PI3K (complex), Ppar, PROX1, RAB23, RAB36, RALGPS2, RGS (2, 4, 7, 16, 18), RGS18, RGS4, RGS7, SH3BP4, SH3RF3, SRGAP2, SYDE1, TFAP2B, YAP/TAZ	35	25	Cancer, Cell Cycle, Organismal Injury and Abnormalities
11	Ahr-aryl hydrocarbon-Arnt, Alpha Actinin, CG, cytochrome-c oxidase, DPT, ERK1/2, EYA1, glutathione peroxidase, glutathione transferase, GPBAR1, GST, GST (complex), GSTA3, Gstt1, GSTT2/GSTT2B, IRF6, ITLN1, LANCL1, MAP3K19, MGST3, MTFR2, NCOA7, PDSS2, PTPase, PTPN14, PTPN4, PTPN7, PTPRK, PTPRN, Ptprv, RGS5, SERPINB5, Sod, TLR7/8, TMEFF2	30	23	Cell Cycle, Drug Metabolism, Glutathione Depletion In Liver
12	AIG1, ARID5A, ARID5B, CCNYL1, CD55, CHST10, CITED2, cNOS, CTSE, ETNK2, Fcer1, GLI2, Hdac, histone deacetylase, IDO, Ifn, IL10, IL23, IRF, KMO, KRTAP12-2, MAN1A1, MEF2, NfkB-RelA, PBX1, PCMTD1, RNA polymerase II, SERPINB13, SERPINB2, SLAMF8, STK36, STX11, Tlr, transcription factor, VAMP4	29	22	Embryonic Development, Endocrine System Development and Function, Protein Synthesis
13	ATP1A2, CACNA1E, Calcineurin A, CaMKII, Ck2, DUSP23, ENPP1, EYA4, FAM162B, Gsk3, HDDC2, IFN Type I Receptor, INPP5D, KIR, L-type Calcium Channel, MFSD4B, MFSD6, MIR124, Na K ATPase, NPAS2, nucleoside-triphosphate diphosphatase, PALD1, PDCD1, Pdgf (complex), peroxidase (miscellaneous), phosphatase, PIKFYVE, PPP1R14C, PPP2R5A, PTPRC, SGPP2, SLC16A10, SMYD3, STAT1, STYXL2	29	22	Hematological Disease, Immunological Disease, Organismal Injury and Abnormalities
14	ACAC, ACBD3, ADAMTS14, BLZF1, c-Src, Collagen type I (complex), Collagen type IV, Collagen(s), cytokine, DNA-methyltransferase, DNMT3L, DYRK3, EPHX1, FHL2, Growth hormone, HBS1L, Histone H2b, HSD11B1, IGFBP2, IGFN1, Integrin, MCU, Metalloprotease, MICU1, NF-Y, NR1I3, Pdgfr, PRRX1, Rab5, RALB, RTN4IP1, SERPINB7, SGK1, STK25, SUCO	27	21	Cell Signaling, Cellular Development, Vitamin and Mineral Metabolism
15	Alpha catenin, ALT, C1orf100, COL3A1, COL5A2, COL6A2, F5, FCER1A, FCER1G, HEY2, Histone h4, ICOSLG/LOC102723996, Ifn gamma, Iga, IgD, Ige, ITGB2, LAMA4, Laminin (family), Mmp, MYCT1, p70 S6k, PECR, PLC gamma, PLD, PRDM1, RGS16, SELL, Serpinb3b/Serpinb3c, SERPINB4, STAT4, SYK/ZAP, Tgf beta, tyrosine kinase, XCL1	25	20	Connective Tissue Disorders, Hematological Disease, Immunological Disease
16	ACADL, AKAP7, BTG2, calpain, CAPN10, CDK5R2, DCAF6, DCAF8, Filamin, Frizzled, GC-GCR dimer, Gli, GLRX2, GPATCH2, Histone H1, K Channel, KCNB2, KCNJ10, KCNT2, LRP, Mir122a, b, Mitochondrial complex 1, MLPH, Mucin, Nr1h, NR5A2, Oxphos, Pkc(s), POU3F3, Rxr, RXRG, SLCO4C1, SYT2, T3-TR-RXR, TRPM2	23	19	Nervous System Development and Function, Organ Morphology, Organismal Development
17	alcohol group acceptor phosphotransferase, APC (complex), Cdk, CDK1, CDK15, CDK18, chemokine receptor, Cyclin A, Cyclin D, Cyclin E, DNA2, E2f, EXO1, FAM20B, FBXO5, KCNH1, KDM5B, LIN9, Mapk, Mcm, MCM3, MCM6, MCM9, NEK2, Neurotrophin, PLAGL1, PP1 protein complex group, Rb, RC, RGS13, RPA, Selectin, SHC2, TSGA10, ZC3H12D	23	19	Cell Cycle, Cell Morphology, Cellular Assembly and Organization
18	1700027J07Rik, AMIGO2, AOX1, APP, ARMC9, C1orf21, C2CD4C, C2orf69, CARF, Cathepsin, CCND1, CXXC4, DCBLD1, farnesyl pyrophosphate, GSTA3, H2-M2, HRAS, ICA1L, IL18RAP, ITM2C, KRAS, LRRN2, MEAK7, MGAT4A, miR-542-3p (miRNAs w/seed GUGACAG), PHACTR2, PTPN1, RASGRP2, RGL1, SLAMF8, SLC6A9, Snhg6, TMCC1, TMEM26, TRIM13	23	19	Cellular Development, Dermatological Diseases and Conditions, Organismal Injury and Abnormalities
19	Adaptor protein 2, ADCY, ADORA1, Calmodulin, Clathrin, CMKLR2, CSTB, estrogen receptor, FZD5, FZD7, G protein, G protein alpha i, G-protein beta, Girk, Gpcr, GPR161, GPR35, GPR37L1, GPR39, GRK, HTR2B, OPRK1, PALM, PDXK, PIK3C2B, PLC, protein phosphatase, PSAP, RAMP1, RAS, Rgs, Secretase gamma, TACR2, TRPA1, voltage-gated calcium channel	21	18	Cell Death and Survival, Cell Signaling, Nucleic Acid Metabolism
20	Actin, ADIPOR1, Arp2/3, CASP8, CFLAR, CHML, collagen type i (family), COPS5, DNPEP, F Actin, GUCD1, HEBP2, Hif1, Ikb, IKBKE, IKK (complex), IKKA/B, KRTAP10-7, Laminin (complex), LBR, MAP1LC3, NCF2, p85 (pik3r), PFKL, PRDX6, RABIF, Rap1, RASSF5, SOAT1, Spectrin, Talin, TSH, tubulin, Ubiquitin, USP40	21	18	Cell Death and Survival, Embryonic Development, Organismal Injury and Abnormalities
21	ADRB, B3GALT2, C8, Camk, CAMK1G, CD3 group, CD48, CNN2, Collagen type II, Cpla2, ERK, G protein alpha, Gi-coupled receptor, GNAZ, HACE1, Igf, Inflammasome (Nalp3, Asc, Casp1), LMOD1, LRRFIP1, MAC, Mettl21e, NMNAT2, Oas, Pde, PDE7B, PLA2G4A, Pln, PRELP, Proinsulin, Ptma (includes others), SMAD1/5, SMPDL3A, TCF, Tropomyosin, ZBED6	20	17	Cell Cycle, Cellular Movement, Hematological System Development and Function
22	20s proteasome, apyrase, arginase, C1Q (family), Cadherin, CDH19, CDH20, CDH23, COL13A1, COL9A1, collagen, Collagen type ix, collagenase, cyclooxygenase, Fcgr3, FH, FMO, FMO1, FMO2, Fmo9, FMOD, G Protein I, GRIK2, H/K/NRAS, Hsp27, MSTN, MYBPH, P38 MAPK, PCDH15, PDGF BB, PFKFB2, Rlc, SESN1, TRAM2, VitaminD3-VDR-RXR	20	17	Auditory Disease, Cellular Movement, Hereditary Disorder
23	AIDA, ARHGEF4, B3GNT7, C/EBP, Collagen type III, CR1L, cytokine receptor, HES6, HINT3, IFNGR, Ikk (family), IL-1R, IL17a dimer, IL17R, Il18r, IL18RAP, IL22RA2, Il8r, IRAK, JAK, Jnk, KANSL3, linear amide carbon-nitrogen bond hydrolase, NIT1, PM20D1, PRKD, Pro-inflammatory Cytokine, SAA, SASH1, Stat3-Stat3, TFCP2L1, Tnf receptor, TNN, VNN1, Vnn3	18	16	Amino Acid Metabolism, Small Molecule Biochemistry, Vitamin and Mineral Metabolism
24	ACMSD, Adaptor protein 1, Ap1, ATF3, C-X-C chemokine receptor, C1q, CCDC28A, CCL20, CD34, chemokine, CXCR/CCR, CXCR2, CYP27A1, Fibrinogen, GPIIB-IIIA, IFN Beta, IFN type 1, IgG, Igm, IL1, IL12 (complex), IL12 (family), IL17F, IL1R1, IL1R2, IL1RL1, INTERLEUKIN, LDL, MAP4K4, NFkB (family), SELE, SELP, SLAMF1, Tnf (family), TRAF3IP2	18	16	Hematological System Development and Function, Immunological Disease, Organismal Injury and Abnormalities
25	ADAT2, AIRE, BRCA1, CADM3, CEP97, COL16A1, CTSA, ELAPOR1, ESR2, HOXC10, ICAM3, ITIH5, KLHDC9, KLHL30, MCMDC2, METTL24, MROH1, NCL, PERP, PGP, POLRMT, PPP1R42, PTPN18, Ptprv, RGSL1, RIGI, RNF122, SAG, SNN, SPOCK2, THOC3, TMCC1, TP53BP2, TP53I11, USH2A	18	16	Cell Morphology, Cell-To-Cell Signaling and Interaction, Cellular Assembly and Organization

**Table 3:** A list of molecular networks of DEGs in CgP versus ReP.

ID	Molecules in Network	Score	Focus Molecules	Top Diseases and Functions
1	ARHGAP18, BETA TUBULIN, c-Src, C1orf226, CCDC115, CCDC6, CCNYL1, DNA damage response, DTL, FAM135A, GJA1, IDH1, ILDR2, MAP7, MICAL1, MSC, NPL, NRP2, OPTC, PCBD1, PCBP3, PDE7B, Plexin A, PLXNA2, SCML4, SEC63, SLC30A1, SOWAHC, ST8SIA4, STAU2, TBC1D8, TMEM169, UBXN4, Vegf, ZBTB2	48	30	Cancer, Dermatological Diseases and Conditions, Organismal Injury and Abnormalities
2	ACTR3, AKAP12, Ant, ARL4C, C2CD4C, CAMSAP2, DAPK, DES, EFHC1, FMO1, FN1, HLX, ING5, KIF21B, LATS1, LYPLA1, Mettl21e, MYL1, NPHS2, Pak, PDGF BB, RASAL2, ROCK, SEMA4C, SH3BP4, SYDE1, SYNE1, TAGLN2, TIMM17A, TNS1, TRPM8, TWIST2, UNC5B, VLDL-cholesterol, WASF1	46	29	Cardiovascular System Development and Function, Cell Morphology, Cellular Movement
3	AIFM2, ASPM, ATP1B1, ATPase, BARD1, CISD1, cytokine, DNA2, ECHDC1, KIF14, MCM3, MCM9, MTHFD1L, NSL1, NUF2, P glycoprotein, PER2, peroxidase (miscellaneous), PHLDA3, PRIM2, Proteasome, PSMD1, RAB36, RAB3GAP1, RABGAP1L, RC, RFTN2, RPA, SPECC1L, TRIP12, UBE2T, XRCC5, YBEY, ZNF365, ZRANB3	43	28	Cancer, Cardiovascular Disease, DNA Replication, Recombination, and Repair
4	C1QL2, Cd24a, CENPL, CNTN2, Collagen type III, Collagen type IV, Cox5b/Gm34962, CSRP1, EFHD1, elastase, ESR1, FAM184A, GPC1, IGF receptor, IGFBP5, Integrinα, LAMA2, LAMA4, LAMB3, LAMC1, LAMC2, Laminin (family), Laminin2, Laminin5, LIMS1, Mmp, PLEKHG1, SERPINB5, Smad, SOX17, STRADB, TCF/LEF, TPD52L1, UCK2, ZBTB18	34	24	Cancer, Organismal Injury and Abnormalities, Reproductive System Disease
5	ADAM23, ADAMTS14, Adaptor protein 2, Alp, ARID3A, CENPW, Collagen type I (complex), COPS5, CPA6, DCAF8, E130307A14Rik, ECEL1, FZD5, GPR39, GPR55, IL1R2, Insulin, Integrin, IRS1, Laminin (complex), Metalloprotease, Osteocalcin, p85 (pik3r), PAPPA2, PERP, PLD, Proinsulin, PRRX1, SATB2, Shc, SLC30A10, SLC39A10, SP110, SP140, SUSD2	32	23	Connective Tissue Development and Function, Connective Tissue Disorders, Organ Morphology
6	Ahr-aryl hydrocarbon-Arnt, BMPR2, CNN2, COL10A1, Collagen type II, COX20, DPT, EPHX1, ERK1/2, F11R, Fibrin, HINT3, HSD11B1, INHA, INHBB, Inhibin, ITLN1, LDL-cholesterol, linear amide carbon-nitrogen bond hydrolase, LRP11, MARCO, NCOA7, Obsl1, PM20D1, Rar, RGS5, SMAD1/5, SMAD1/5/9, Stat3-Stat3, TCF, TLR7/8, TRPM2, TUBE1, VNN1, Vnn3	30	22	Amino Acid Metabolism, Small Molecule Biochemistry, Vitamin and Mineral Metabolism
7	alcohol group acceptor phosphotransferase, Ap2, Ap2 alpha, apyrase, atypical protein kinase C, CD84, Ciap, Clathrin, EDAR, FAM20B, FBXO30, GULP1, Il12 receptor, Ku, LSS, LY9, MTFR2, MYBL1, Na K ATPase, NEK2, Nfatc, NFkB (complex), NUAK2, PEX19, PNKD, RALGPS2, RGS18, RNF152, RTKN2, SH2D1B, SLC19A2, SLC2A12, ST18, TFAP2B, TRAF	30	22	Cell Death and Survival, Dermatological Diseases and Conditions, Developmental Disorder
8	ANK3, APC (complex), calpain, CENPF, CHCHD10, CYB5R1, Cyclin B, cytochrome-c oxidase, DERL3, EIF4EBP2, ENAH, FBXO5, GC-GCR dimer, GLRX2, glutathione peroxidase, Gstt3, HBS1L, HJURP, ion channel, IVNS1ABP, LIN9, Mapk, Mitochondrial complex 1, MLPH, MPZ, NDUFS2, NFASC, Oxphos, PDSS2, PLAGL1, Rab5, RGS13, Selectin, SPTA1, voltage-gated sodium channel	30	22	Cellular Assembly and Organization, Cellular Function and Maintenance, Nervous System Development and Function
9	ALT, BCR, C1orf112, Ck2 alpha, DIP2A, EGR2, FASLG, GOT, hemoglobin, HLA-DR, IgD, IL18R1, INPP5D, Interferon alpha, MAPKAPK2, MHC CLASS I (family), MHC Class II (complex), MHC II, NAB1, NABP1, PARD3B, PDCD1, PRDX6, PRF1, protein phosphatase, RAMP1, RCSD1, RNF126, SLAMF7, SLC35D3, STAT4, STAT5a/b, TMCC2, UBE2D1, Ubiquitin	30	22	Cardiovascular Disease, Cell Death and Survival, Gastrointestinal Disease
10	ATP1A2, Calcineurin A, CD48, Ck2, DUSP10, DUSP23, DUSP28, ERK, EYA1, EYA4, FH, Gi-coupled receptor, Gsk3, HDDC2, IFN Type I Receptor, Igf, Igfbp, Na, K -ATPase, NMDA Receptor, Nos1ap, PALD1, Pdgf (complex), phosphatase, PIKFYVE, PP1 protein complex group, PPP1R14C, PTP4A1, PTPase, PTPN18, PTPN7, PTPRC, PTPRK, PTPRN, Ptprv, STYXL2	28	21	Hematological Disease, Immunological Disease, Post-Translational Modification
11	26s Proteasome, ADRB, ATG4B, BCL2, BCR (complex), BLZF1, BOK, caspase, CDK18, Cyclin A, cytochrome C, DSTYK, DYRK3, EXO1, FBXO28, FOXO3, H/K/NRAS, HISTONE, HSD17B7, Ifn gamma, MAP3K5, MIR101, PARP, PKP1, PP2A, Ppp2c, PRKAA, REV1, SLAMF1, STEAP3, TMEM9, TP53BP2, TRAF5, TSGA10, XPR1	28	21	Cell Cycle, Cell Death and Survival, Organismal Injury and Abnormalities
12	ACMSD, ACTR1B, BVES, C-X-C chemokine receptor, CCL20, collagen type i (family), CXCR/CCR, CXCR2, CXCR4, DOCK10, GLUL, GTPase, HDL, Hif1, Ifn, IFN Beta, IL12 (family), IL17A, IL17F, LDL, MRPL44, NFkB (family), NGEF, Notch, PI3K (family), PLEKHB2, RNA polymerase II, RNASEL, Rnr, SELP, SMYD3, TNFAIP3, TRAF3IP2, TRAF3IP3, UTP14C	26	20	Hematological Disease, Immunological Disease, Organismal Injury and Abnormalities
13	14-3-3, AGPAT3, AIG1, ATIC, BSG, Calcineurin protein(s), CASP8, CDK1, CEP170, CXCR1, GPR35, GPR37L1, HDL-cholesterol, Histone h2a, Histone h3, Histone h4, IFI16, Ikb, IKK (complex), IKKA/B, ITPR, LANCL1, LPGAT1, MAP2K1/2, NDUFA10, NDUFB3, NDUFS1, Nfat (family), PFKL, Pka catalytic subunit, PLC gamma, PTK, SOAT1, THAP4, TSPAN15	26	20	Developmental Disorder, Hereditary Disorder, Metabolic Disease
14	Akt, ANGPTL1, B3GAT2, BTG2, Cdk, CHST10, CHST3, Collagen type VI, CREG1, Cyclin E, death receptor, DNA-PK, E2f, FCGR1A/2A/3A, Fgf, FGFR, Foxo, INPP4A, Integrin alpha 6 beta 4, LMBRD1, mir-205, N-Cadherin, NYAP2, PALM, PAM, Pki, RGS8, SEC16B, SERCA, SLC9A2, SULF1, sulfotransferase, SUSD4, UHMK1, UST	24	19	Carbohydrate Metabolism, Connective Tissue Development and Function, Skeletal and Muscular System Development and Function
15	Alpha Actinin, arginase, C1Q (family), Cadherin, CASQ1, Cbp/p300, CDH19, CDH23, CNNM4, COL13A1, EFCAB2, estrogen receptor, FMN2, Hdac, histone deacetylase, Histone H2b, IRF, IRF6, KCNE4, KCNQ5, KIF1A, LONRF2, MEF2, MYOG, N-cor, NF-Y, P38 MAPK, PFKFB2, PLEKHM3, Rb, succinate dehydrogenase, TNNI1, transcription factor, TRDN, ZC3H12D	23	18	Cancer, Organismal Injury and Abnormalities, Skeletal and Muscular Disorders
16	AHI1, ARID5A, beta-galactosidase, CD55, cNOS, COL5A2, COL6A3, collagen, collagenase, CRYBA2, CTSE, DDT, GGT1, GPIIB-IIIA, Growth hormone, Hsp27, Hsp70, IL1, IL10, JINK1/2, JUN/JUNB/JUND, LAD1, LCT, NfkB-RelA, NfkB1-RelA, Ngf, Rxr, RXRG, SELE, SERPINE2, Smad2/3-Smad4, STX11, Tnf (family), TRAM2, VAMP4	23	18	Inflammatory Disease, Organismal Injury and Abnormalities, Protein Synthesis
17	Alpha 1 antitrypsin, BPNT1, CDK15, chemokine receptor, Cpla2, CRYGA, CSF, Defensin Alpha, Ecm, EGLN, ELANE, growth factor, IgG2c, K Channel, KCNB2, KCNT2, LRP, NADH dehydrogenase, NADPH oxidase, NEURL3, Neurotrophin, Par, Pkc(s), PPOX, PREP, PRSS57, PRTN3, QRSL1, Serine Protease, Serpin, SERPINB8, SHC2, SLCO4C1, Srgn, SUCO	21	17	Cell-To-Cell Signaling and Interaction, Dental Disease, Dermatological Diseases and Conditions
18	Adaptor protein 1, CD247, CD28, CD3, CD3-TCR, CD8, CD80/CD86, chemokine, Cofilin, CTLA4, DISC, FYN, GTF3C6, HCN2, ICOS, IL-2R, IL-35, Integrin alpha L beta 2, KIAA0408, LBR, LCK/Fyn, MAP2K4/7, NCK2, NCKAP5, NFAT (complex), NIT1, Pde4, PPA1, RNPEP, SAG, SLAMF6, SPATC1L, TCR, TH1 Cytokine, TH2 Cytokine	21	17	Hematological System Development and Function, Lymphoid Tissue Structure and Development, Tissue Morphology
19	ADCY, Beta Arrestin, CACNA1E, Calmodulin, CAMK1G, CaMKII, CHPF, DNER, G protein, G protein alpha, G protein alpha i, Girk, GNAZ, Gpcr, GRK, KIR, MCU, MFSD4B, MFSD6, OPRK1, Pdgfr, PIK3C2B, PLC, RALB, RAS, Rgs, RGS17, Rsk, RTN4IP1, Secretase gamma, SLC16A10, STAT, TOR1AIP2, TRPA1, voltage-gated calcium channel	19	16	Cell Signaling, Molecular Transport, Nervous System Development and Function
20	Alpha catenin, Ap1, ARPC5, C1q, CD34, CG, CK1, Collagen(s), Creb, Fibrinogen, FSH, GNRH, HHAT, HTR2B, IFN alpha/beta, IFN type 1, KANSL3, Lh, LMAN2L, MIF, MYB, MYCT1, Nos, p70 S6k, Pka, PPP1R15B, PTGS2, Ras homolog, RGS2, SGK1, SOBP, TET1, Tgf beta, TMEM163, Wnt	19	16	Cellular Development, Cellular Growth and Proliferation, Hepatic System Development and Function
21	ACAA1, ADARB1, ARMC2, ATP5MC2, BICC1, CADM3, chondroitin sulfate E, CTNNB1, DDX24, FGFR2, Foxo, H60b/H60c, HSD17B7, IGFBP2, LEFTY2, LGR6, miR-132-3p (and other miRNAs w/seed AACAGUC), miR-18a-5p (and other miRNAs w/seed AAGGUGC), NCL, PAQR8, Pbsn, PI3K p85, PLPPR4, PPP1R42, PRL, PTEN, PTGFR, RFX8, Rpl36a, SDHC, SLC30A10, SNED1, STOX1, UPB1, Wfdc18	19	16	Cancer, Organismal Injury and Abnormalities, Reproductive System Disease
22	AATK, AFF3, ASTN1, CD37, CD84, CDH17, CLEC1B, complement receptor, CTSE, Dnm3os, E330020D12Rik, ELAVL1, FKHR, FOXO1, GAB1, Grb2-Shc1-Sos, IGF1R, LACTB2, LAMP1, LGR6, mir-139, miR-139-3p (miRNAs w/seed GGAGACG), PCMTD1, SBSPON, SLC18B1, SLC23A3, SLC25A23, STAT5A, SYK, TBCB, TCF7L2, TFAP2B, TNFRSF11A, TRAK2, VPREB3	19	16	Embryonic Development, Hematological System Development and Function, Humoral Immune Response
23	ACSL3, C8, CD3 group, cyclooxygenase, DDR2, EPHA4, Fcer1, Fcgr3, Gli, GLI2, Gα12/13, IGFBP2, IL23, Inflammasome (Nalp3, Asc, Casp1), KCNJ10, KMO, LATS, MAC, Marcks, Mir122a, b, Mucin, Muscarinic cholinergic receptor, MYBPH, Nr1h, PI3K (complex), PLA2, PLA2G4A, PRELP, Rlc, RSPO3, SCCPDH, STK36, Tlr, YAP/TAZ, ZAP70	17	15	Lipid Metabolism, Molecular Transport, Nervous System Development and Function
24	CCL27, COL15A1, COL16A1, COL6A1, COL6A2, Col6a4, COL6A5, COL6A6, Cxcl15, DDO, DEFB125, ENPP3, FUCA2, HEY2, IGFL1, Il8r, ITGA10, METTL24, OLFML2B, PEX5, PIM3, POFUT2, RAB37, RAMP1, RCN3, RGSL1, RIGI, SLC11A1, SLC19A3, ST3GAL6, TNF, TREML1, UMOD, USP40, XCL1	17	15	Cancer, Connective Tissue Disorders, Dermatological Diseases and Conditions
25	ABCA3, ACTR2, ARHGAP30, ATG2B, ATP6V1H, CEP85L, CISD1, CRIP1, DDX59, DOCK7, ENO1, EYA4, FBXO36, GPR107, ITPRIPL1, KANSL1L, KLHDC9, LRCH4, LRRN2, LYST, MICAL2, MYCN, OPTN, PPIP5K2, Rps3a1, RRP7A, SLC9A4, STING1, SYNE1, TBC1D17, THOC3, TMEM63A, TOMM40L, TRAF2, ZFAND6	17	15	Cardiac Dilation, Infectious Diseases, Organismal Injury and Abnormalities

### 3.4 DeP and CgP both showed suppression of immune system processes

**Figure 4:**
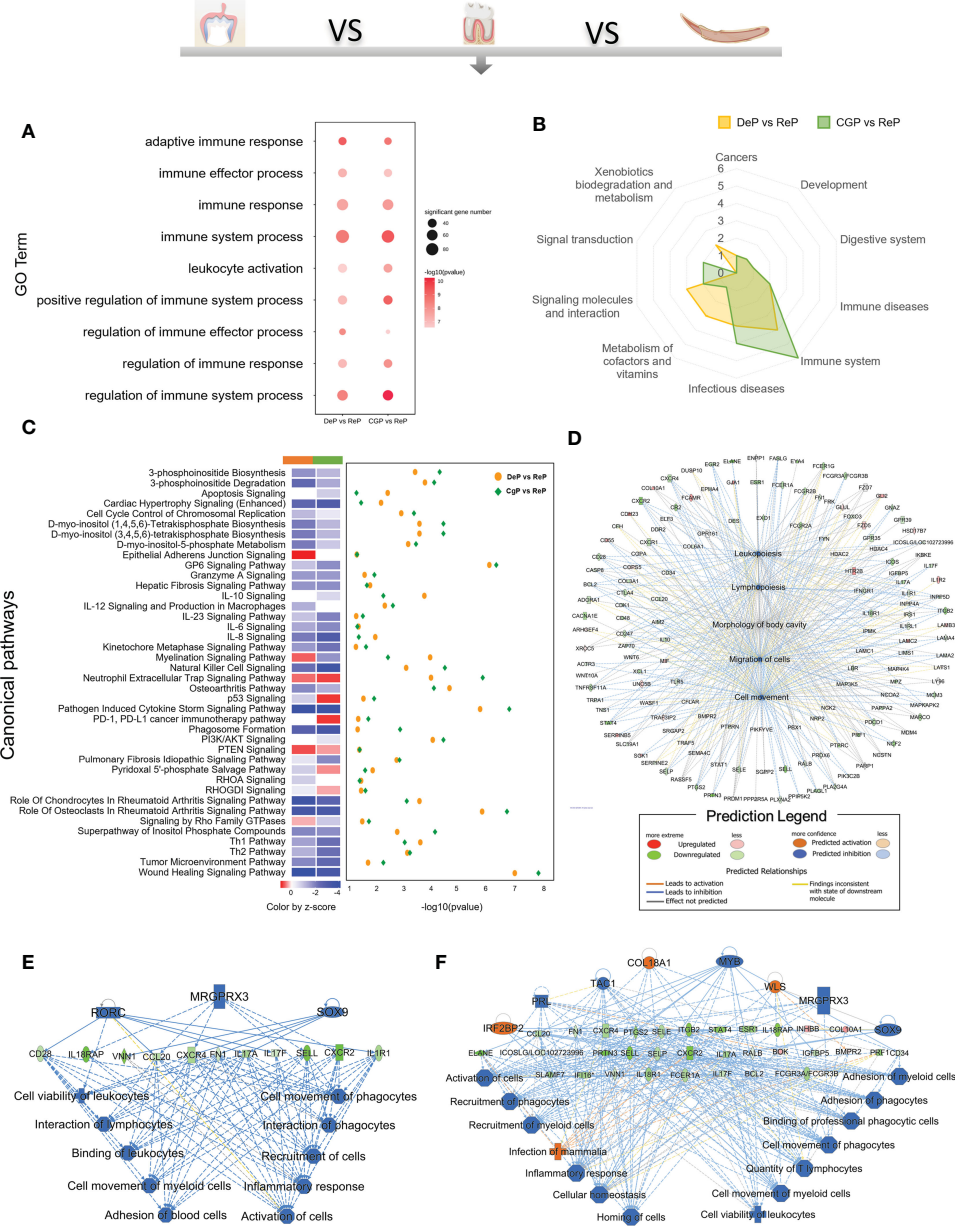
GO, KEGG database enrichment analyses, and IPA analysis were used to determine the critical function of the downregulated DEGs in DeP and CgP. **(A)** In the GO enrichment analysis of downregulated DEGs, immune system-related pathways in the top 20 pathways of DeP and CgP. **(B)** Radar map of the classification of the top 20 pathways in KEGG enrichment analysis of DeP and CgP. **(C)** The common canonical pathways enriched by DeP and CgP DEGs in IPA analysis. The z-score of the pathway is shown in the heat map, and the -log10 (p-value) of the pathway is shown in the scatter map. **(D)** “disease and function” predictions were made based on DEGs involved in common canonical pathways. The figure shows the top five functions. **(E, F)** Network diagram representing the regulatory effects with top consistency scores in DeP **(E)**/CgP **(F)** versus ReP.

Enrichment analysis of the downregulated DEGs in the DeP and CgP compared with the ReP showed that the top 20 biological processes, according to p-value, accounted for the most significant proportion in the biological processes related to immune response regulation (7/20). **Figure 4A** shows the immune system processes enriched in the top 20 biological processes, including adaptive immune response, immune effector process, immune response, immune system process, leukocyte activation, positive regulation of immune system process, regulation of immune effector process, regulation of immune response, and regulation of immune system process. Consistent with this, in the KEGG analysis, both showed prominent downregulation in the pathways classified in the immune system, as shown in **Figure 4B**, involving the differentiation of immune cells such as Th17 cells, T cell receptor signaling pathway, and immune cell-mediated cytotoxicity. These results showed that there are immune-mediated tissue characteristics in both the DeP and CgP.

The IPA system showed that the DEGs in the DeP versus the ReP and CgP versus ReP were enriched in multiple canonical pathways. Overall, 93 and 101 canonical pathways were identified by applying p < 0.05 as the threshold. **Figure 4C** shows the p-value and z-score of the 40 common classical pathways that were predicted. Functional predictions of DEGs involved in these common pathways showed that the functions of the top five involved leukopoiesis, lymphopoiesis, morphology of body cavity, migration of cells, and cell movement (**Figure 4D**).

Furthermore, the upstream regulatory network and downstream functions involved in DEGs were predicted by regulatory effect analysis. The consistency score evaluates the causal consistency of upstream regulators in the network, DEG datasets, and dense connectivity measures between disease and function. Fifty-four regulatory effects were identified in the DEGs of the DeP versus the ReP. Among them, the highest-ranking regulatory effect had a consistency score of 31.056, which strongly predicted that *Mrgprx3*, *Rorc*, and *Sox9* in the DeP might participate in the downregulation of the movement and interaction of lymphocytes, myeloid cells, and phagocytes by mediating their targets (**Figure 4E**). Meanwhile, 47 regulatory effects were identified in the DEGs of CgP versus ReP. The highest ranked regulatory effect with a consistency score of 35.355 strongly suggested that the regulators *Col18a1*, *Irf2bp2*, *Mrgprx3*, *Myb*, *Prl*, *Sox9*, *Tac1*, and *Wls* may participate in extensive regulation of cellular behavior in immune processes (**Figure 4F**).

These results suggested that the altered gene expression was closely related to the immune response in the DeP and CgP. There were apparent differences in the status of leukocytes and myeloid cells in the tissues, which may represent their common tissue characteristics during active tissue remodeling.

### 3.5 There were differences in the regulatory genes involved in tissue formation between the DeP and CgP

**Figure 5:**
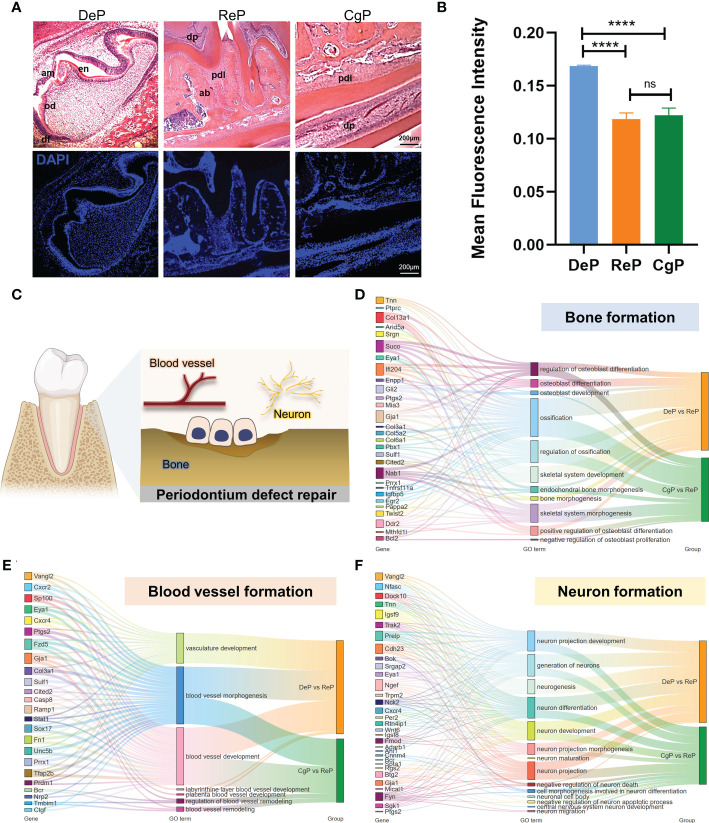
Comparison of the characteristics of tissue formation processes. **(A)** H&E staining and immunofluorescence staining of dental follicles and periodontium in mouse mandibular in sagittal sections. **(B)** The mean fluorescence intensity of DAPI was used to represent the cell density in the tissue. ****p < 0.0001. **(C)** Diagram of the formation of several vital tissues during periodontal remodeling. **(D–F)** Sankey maps are used to show critical genes and pathways involved in bone, blood vessel, and neuron formation. ns, no significance.

To further explore tissue characteristics, tissues from the DeP, CgP, and ReP were prepared as tissue sections and subjected to H&E staining, as shown in **Figure 5A**. The mean DAPI fluorescence intensity was used to represent the live cell density in the tissue. The cell density in the DeP was significantly higher than that in the other two groups, which may be related to the threshold characteristics in early development (**Figure 5B**). During remodeling, periodontal cells can differentiate and participate in the formation of different tissues. Promoting the formation of bone, blood vessels, and nerves is an essential basis for functional periodontium regeneration (**Figure 5C**).

Since DeP and CgP have different characteristics in the tissue microenvironment, we summarized the significant genes (top 20 based on q value) involved in cell differentiation in these two tissues to better understand and apply their regulatory targets. As shown in **Figure 5D**, *Suco, Ifi204, Gja1, Col13a1*, and *Nab1* were involved in the most extensive bone formation pathways compared with other genes and may be the core genes in the process of osteoblast differentiation and ossification in periodontal tissue. As for blood vessel formation, most genes showed enrichment in blood vessel development and morphogenesis. We noticed that only the DEGs of DeP versus ReP were enriched in the vasculature development pathway, while the blood vessel remodeling process was explicitly enriched in CgP versus ReP. *Tmbim1* was a specific gene enriched during blood vessel remodeling (**Figure 5E**). In the formation of nerve tissue, the significant genes of the two differed. Only nine genes, *Bok, Cdh23, Eya1, Igsf9, Ngef, Prelp, Trpm2*, and *Vangl2*, were significantly different (**Figure 5F**). It can be seen that there were differences in the regulatory genes involved in tissue formation between the DeP and CgP.

### 3.6 Key regulatory signals of DeP and CgP tissues

**Figure 6:**
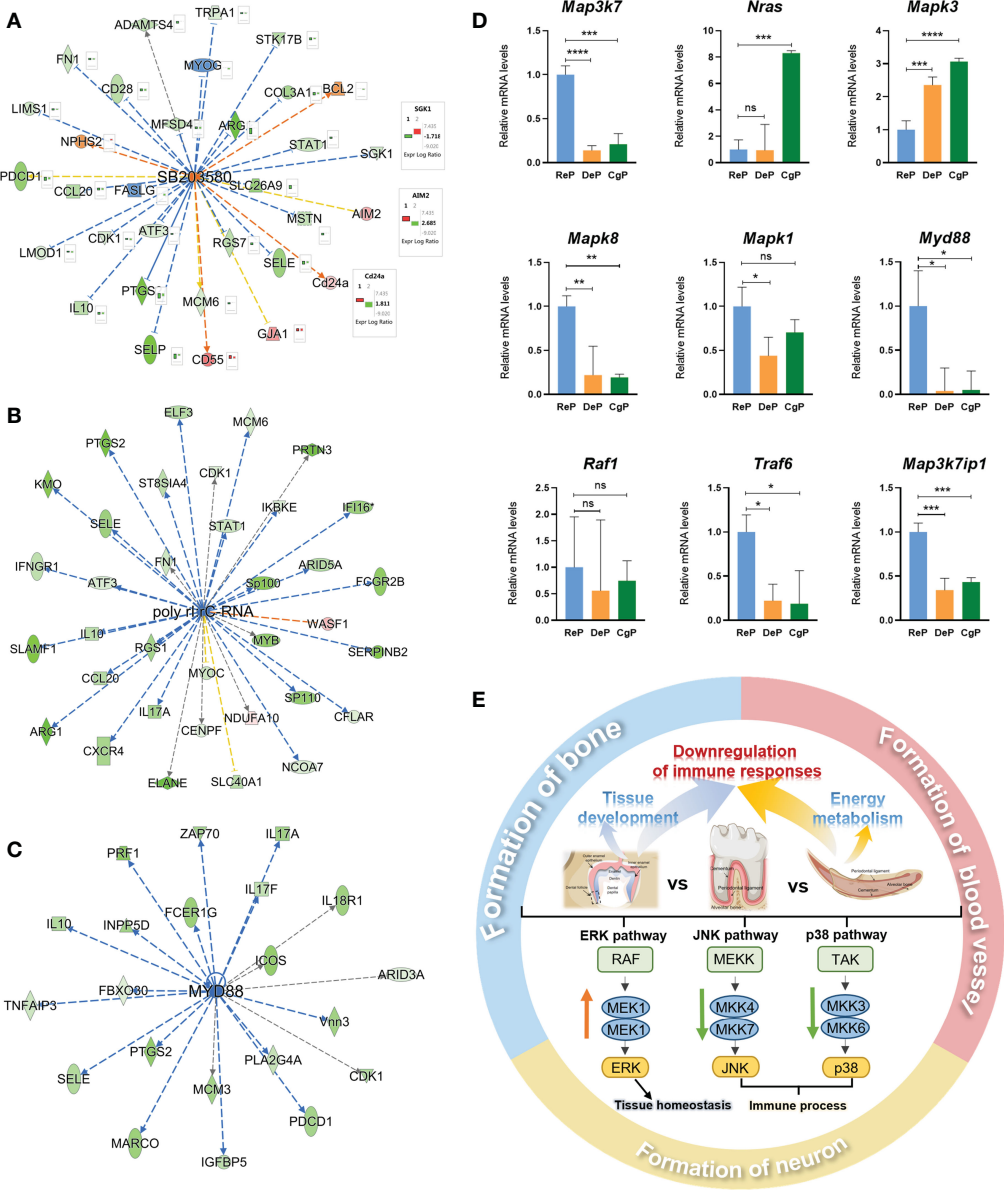
Prediction of upstream regulators and validation of target genes. **(A–C)** Network diagram representing the upstream regulators with highest and lowest z-scores. **(D)** mRNA expression of 9 representative genes was validated using RT-qPCR. *P < 0.05, **P < 0.01, ***P < 0.001, ****P < 0.0001. **(E)** Schematic diagram of active remodeling periodontal tissue characteristics. ns, no significance.

To further explore the possible key regulatory signals during periodontal remodeling, the upstream regulators and potential associations of 795 and 675 DEGs in the two comparisons were analyzed. By applying the p-value of overlap < 0.05 threshold, 1637 upstream regulators were enriched in the DeP versus ReP, of which 65 regulators with a z-score > 2 were predicted to be activated and 173 with a z-score < −2 were predicted to be inhibited. Under the same threshold, a total of 1986 upstream regulators were enriched in the CgP versus ReP, of which 59 regulators were expected to be activated, and 135 regulators were predicted to be inhibited. The chemical drug SB203580 was the most potent activator common to both groups (Z-score = 3.798 in DeP versus ReP, and Z-score=3.405 in DeP versus ReP). **Figure 6A** shows its 29 target molecules enriched in the two groups of DEGs. The expression trends of other genes showed consistency between the two groups except for *Skg1*, *Aim2*, and *Cd24a*. In addition, poly I:rC-RNA (Z-score = −4.537, **Figure 6B**) and MYD88 (Z-score = −3.897, **Figure 6C**) were the most potent inhibitors in the DeP versus ReP and the DeP versus ReP, respectively, regulating the expression of 34 and 21 target molecules in periodontium remodeling.

SB203580 is an inhibitor of the p38 MAPK pathway (15). Poly rI:rC-RNA effectively promotes the phosphorylation and activation of MAPK pathway molecules (16), while the adaptor protein encoded by Myd88 transduces Toll-like receptor signals and activates NF-κB and MAPK pathways (17). The activation and inhibition status of these molecules as upstream regulators strongly suggested that the MAPK pathway was inhibited in the DeP and ReP and may be closely related to the periodontium remodeling process. In total, nine genes involved in the MAPK pathway were selected for qPCR validation. The genes that were upregulated in the actively remodeling periodontium were *Mapk3* and *Nras*, and the downregulated genes were *Mapk8, Mapk1, Map3k7, Myd88, Raf1, Traf6*, and *Map3k7ip1* (**Figure 6D**).

## 4 Discussion

Exploring the mechanism of postnatal tissue development and adult regeneration is significant for understanding tissue regeneration. To improve clinical therapeutic outcomes of periodontium defects, it is necessary to understand the physiological process of periodontal remodeling in both the developmental and adult stages. In postnatal mice, the dental follicle structure forms the periodontal tissue, which regulates bone remodeling and tooth eruption. Unlike molars, mice incisors grow continuously throughout life, completing a renewal every 35–45 days (18). The mature mandibular incisor is attached to the alveolar bone only on the lingual side through the periodontal ligament tissue. Studies showed that collagen remodeling was more active on this site, making mice an ideal model for periodontal tissue formation and continuous remodeling. Here, we isolated tooth follicles and lingual periodontal tissues of the incisor, and compared them with periodontal tissue of the fully erupted molar to explore the tissue characteristics and critical mechanisms involved in the active remodeling process of periodontal tissues with different temporal and spatial distributions.

### 4.1 DEGs in the DeP/CgP versus ReP

Our study identified 792 and 612 DEGs in the DeP and CgP compared with the ReP. The Venn diagram results shown in **Figure 2E** suggested that gene regulation in the DeP and CgP was generally similar but had certain distinct features. These different molecular features emphasized that the remodeling activities occurring in developing and adult periodontium can inspire the repair and regeneration of periodontal tissues from different perspectives.

### 4.2 DeP and CgP showed different upregulated biological processes during periodontal remodeling

The pathway analysis suggested that upregulated DEGs in the DeP/CgP versus the ReP were enriched in various biological processes in developmental and adult stages during the active remodeling of the periodontium. The DeP showed enhanced activities related to tissue development. These results are highly consistent with previous studies showing that the periodontal tissue undergoes a developmental process from the beginning at the dental follicle stage (19). The dental follicle is a loose ectoderm-derived connective tissue surrounding the tooth germ that can differentiate into cementoblasts and secrete substances to form root cementum. At the same time, dental follicle cells secrete collagen fibers in the newly generated cementum to fix the tooth root in the alveolar fossa to form the root–bone interface and coordinate tooth eruption (20). At this stage, tooth morphogenesis is active.

Interestingly, unlike classical signals in tooth development, upregulated dental follicle DEGs in the KEGG analysis showed significant enrichment in the Hippo signaling pathway, as shown in **Figure 3C**. It has been confirmed that the critical effectors of the Hippo pathway, Yes-associated protein (YAP) and its homolog PDZ-binding motif transcriptional coactivator (TAZ), enhance the stability and nuclear translocation of β-catenin and participate in the regulation of osteogenic and adipogenic differentiation of oral stem cells (21). Multiple studies have shown that Hippo signaling regulates the proliferation, differentiation, apoptosis, and senescence of periodontal ligament stem cells and the differentiation and mineralization of cementoblasts through crosstalk with the ERK1/2 pathway, ROCK pathway, TGF-β/BMP pathway, Wnt/β-catenin pathway, and BCL-2 (22). Our results confirmed that the dental follicle provides the reference for the orderly generation of periodontal tissue from scratch, and Hippo signaling is an essential regulator of periodontium development.

Just as important, the processes involved in energy metabolism were the most prominent in the enrichment analysis of the upregulated DEGs in the CgP versus ReP, especially aerobic metabolic processes, such as oxidative phosphorylation and the TCA cycle, indicating that significantly different metabolic characteristics accompanied the active remodeling state in adult periodontal tissues. Indeed, in addition to growth factors and extracellular matrix signaling, there is increasing evidence that various metabolic pathways provide essential signals for the self-renewal and differentiation capacity of cells, thereby regulating the epigenome and influencing cell fate (23). The critical link of periodontal tissue remodeling is the synthesis stage of osteoblast differentiation, which requires a large amount of energy (24). The process of mitochondrial phosphate oxidation is an efficient way to generate energy. A dynamic proteomic analysis of human periodontal ligament stem cells during osteogenic differentiation showed that oxidative phosphorylation was the most critical regulatory pathway during differentiation (25), and oxygen tension significantly regulated the activity of alkaline phosphatase in periodontal ligament fibroblasts (26). The inhibition of osteoblast differentiation in the aged periodontal ligament may be directly related to the difficulty in completing cellular respiration (27). Our results suggested that metabolic status may represent the critical mechanism during the remodeling process of the adult periodontium, and promoting periodontal remodeling and osteogenic repair processes based on metabolism regulation may be a promising approach for periodontium regeneration.

### 4.3 Comparison of the tissue formation processes in developmental and adult stages

The aim of periodontal regeneration therapy includes the regeneration of hard and soft tissues. Through the gene analysis of bone, blood vessels, and neurons, we summarized the gene regulation paradigm, which can be introduced into animal models for more in-depth research to provide more clues to the biological events of periodontal reconstruction. Among the most significant DEGs, *Suco, Ifi204, Gja1, Col13a1*, and *Nab1* most extensively played roles in bone formation-related biological processes. Among them, only *Ifi204*, the murine homolog of human *IFI16*, showed consistent inhibition of expression in the DeP and CgP, which regulates the release of IL-1β in tissues and plays a vital role in bone loss in experimental periodontitis. *Suco, Gja1, Col13a1*, and *Nab1* were significantly upregulated. These genes have been widely studied in the regulation of osteoblast differentiation and bone remodeling (28–30), but their roles in regulating periodontal bone health are rarely reported. The concordant results between the DeP and CgP suggested the expression paradigm of these critical genes during alveolar bone remodeling. The combinatorial regulation of these essential genes to promote alveolar bone regeneration is worth exploring.

During the biological process of blood vessel formation and remodeling, although active blood vessel development occurs in both tissues, we noticed CgP-specific enrichment in the process of blood vessel remodeling. This process is different from vasculogenesis, in which angiogenic progenitor cells migrate to sites of vascularization, differentiate into endothelial cells, and coalesce to form the initial vascular plexus, and instead is a remodeling process of existing blood vessels (31). Blood vessel remodeling in the CgP may be related to adapting to the characteristics of the tissue microenvironment, such as precisely regulating O_2_ delivery to maintain oxygen homeostasis and meeting the energy metabolism demand in the periodontal tissue (32). Therefore, it is suggested that attention to the characteristics of the microenvironment of the erupted tooth tissue, such as the microstructural changes of blood vessels caused by chewing force, is of great significance to understanding the biological mechanism of blood vessel formation and remodeling to promote good blood circulation during periodontal regeneration. As there is a complex neural network in periodontal tissue, we also analyzed the nerve formation process. The local sensory and sympathetic nerves play an essential role in developing the dental follicle, maintaining periodontal health, and promoting local disease recovery (33). The abundant enrichment of related genes, shown in **Figure 5F**, suggests that different molecular guidance cues may exist for innervation in developing and mature periodontal tissues.

### 4.4 Characteristics of the immune microenvironment in the DeP and CgP

In terms of commonalities, the tissue characteristics of the DeP and CgP were surprisingly highly correlated with the immune system. In both of them, the activation, migration, and recruitment of immune cells (such as lymphocytes), the production of inflammatory factors (such as TNF and IL17), and the signaling of immune cells (such as the T cell receptor signaling pathway) were inhibited relative to the ReP. This result suggested that unique immune cell activities and cytokine levels may contribute to the characteristics of actively remodeling periodontium. There may be different reasons for this characteristic. First, there is consideration of the unique environment of periodontal tissue and its proximity to oral plaque biofilms. The oral microbiota induces many immune cells to generate periodontal immune responses due to the highly permeable junctional epithelial attachments on the tooth surface (34). Therefore, microbial biofilms in healthy individuals can significantly mobilize the periodontal immune microenvironment and mediate the maintenance of periodontal homeostasis (35). However, the dental follicle structure of the unerupted tooth and the periodontal tissue of the middle part of the incisor is relatively distant from the oral microbial community and show relatively silent immune activities. Second, there may have been an immunosuppressed population of cells in both tissues. Studies have shown that cytokines secreted by dental follicle cells inhibited lymphocyte proliferation (36), and osteoprotegerin secreted by dental follicle cells inhibited monocyte recruitment and osteoclast formation (37). Despite the need to form an osteoclastic resorption channel during tooth germ development and eruption, this seems consistent given the simultaneous alveolar bone formation.

At the same time, stem cell niches exist in the root of the mouse incisor as a cell population that inhibits inflammation and immune regulation, which may regulate the immune activity of the periodontal tissue of the incisor (38). Our results suggest that the actively remodeling periodontium has unique immune system characteristics in both developmental and adult stages. The tissue microenvironment characteristics may regulate and, in turn, coordinate the tissue remodeling process.

### 4.5 Possible regulatory role of MAPK pathway in periodontal remodeling

Further analysis of upstream regulators predicted that SB203580, poly rI:rC-RNA, and Myd88 were identified as potent regulators that might have significant regulatory effects on gene expression in actively remodeling periodontium. Unexpectedly, all three molecules individually suggested an inhibitory state of the mitogen-activated protein kinase (MAPK) pathway. MAPK is a highly conserved serine/threonine protein kinase in eukaryotes. It starts from the activation of upstream MAP kinase kinase kinase kinase (MKKK) (39). Eventually, it leads to the cascade activation of the pathway by targeting a series of downstream substrate proteins for phosphorylation. Three MAPK families have been characterized: the classical MAPK (also known as ERK), C-Jun N-terminal kinase/stress-activated protein kinase (JNK/SAPK), and p38 MAPK. As the MAPK signal transduction cascade plays a critical regulatory role in cell proliferation, differentiation, development, inflammatory response, and apoptosis (40), here we speculated that regulation of the MAPK pathway might be an essential factor in the microenvironment characteristics of the DeP and CgP and further modulates the cellular behavior of the remodeling process. We next selected genes of interest in the pathway for qPCR validation. The genes (*Mapk3* and *Nras*) involved in the ERK pathway showed upregulated levels in DeP and CgP, while the genes involved in the JNK and p38 MAPK pathways (*Mapk8, Mapk1, Map3k7, Myd88, Raf1, Traf6*, and *Map3k7ip1*) were downregulated.

The expression trends of these genes showed consistency with previous studies. It has been proven that different extracellular stimuli can preferentially activate different MAP kinases, thus producing corresponding effects. Growth factors (41) and G protein-coupled receptors (42) can activate the ERK pathway and regulate cell proliferation and differentiation. Active tissue formation occurs in DeP and CgP, which may be related to the activation of the ERK pathway in response to growth factors. Meanwhile, inflammatory cytokines (such as IL-1 and TNF-α) and many cellular stress-inducing factors preferentially activate JNK/SAPK and p38 MAPK (43). Pathogen stimulation in oral biofilms is essential in activating the p38 MAPK pathway in periodontal tissue (44). The unique anatomical features of DeP and CgP reduce the activation of this pathway by inflammatory cytokines and may further regulate the characteristics of the immune microenvironment of these two groups. However, the activation of pathways by stimuli is not one or the other, and studies have demonstrated that the MAPK signaling pathway plays a complex regulatory role in periodontal regeneration. The activation of the ERK/p38 MAPK pathway is involved in the process of osteogenic differentiation of periodontal ligament cells stimulated by asarylaldehyde, but the JNK pathway is not activated (45).

On the other hand, the same pathway may play opposite roles during cell differentiation under different microenvironment signals. Pharmacological antagonism of the p38 MAPK pathway can effectively inhibit the tissue destruction process of periodontitis and promote bone regeneration (46, 47). In a word, as the MAPK pathway can regulate the regeneration of periodontium, the exploration of its key targets and corresponding stimulating factors is of great significance. Combining upstream regulators with gene expression levels, the MyD88/p38 MAPK pathway may be inhibited in the DeP and CgP, and the levels of other MAPK-related pathways in actively remodeling periodontium were altered varied according to the functional differences of each pathway.

**Figure 6E** shows the schematic diagram of actively remodeling periodontal tissue characteristics. The developmental periodontal tissue was enhanced in processes related to tissue development, and the enhanced energy metabolism is characteristic of the adult actively remodeling periodontium. Both stages showed a decrease in the immune response. The MAPK pathway may play an essential regulatory role in microenvironment features and be involved in tissue formation.

## 5 Conclusion

The developmental periodontium and continuously growing periodontium, which are actively remodeled, have shared and unique differences in microenvironmental tissue characteristics and molecular regulatory mechanisms, such as the upregulation of tissue development and metabolic state and common inhibition of the immune system. The MAPK pathway (especially MyD88/p38 MAPK) may be a promising therapeutic target to promote periodontal remodeling. This study provides a deep physiological understanding for promoting the orderly regeneration of the complex structure of periodontal tissue by considering the characteristics of tissue and microenvironment and providing a basis for selecting targets for regenerative therapy.

## Data availability statement

The datasets presented in this study can be found in online repositories. The names of the repository/repositories and accession number(s) can be found below: https://www.ncbi.nlm.nih.gov/bioproject/PRJNA928506.

## Ethics statement

The animal study was reviewed and approved by the Laboratory Animal Ethics Committee in Ninth People’s Hospital Affiliated to Shanghai Jiao Tong University School of Medicine.

## Author contributions

YD and NL analyzed the data and drafted the manuscript. MX performed the dissection and processing of the tissue samples. LH helped the conception of this study. RJ and NH reviewed and revised the manuscript. JW and XJ contributed to the concept and design of the study. All authors contributed to the article and approved the submitted version.
